# Segmentation of epidermal tissue with histopathological damage in images of haematoxylin and eosin stained human skin

**DOI:** 10.1186/1471-2342-14-7

**Published:** 2014-02-12

**Authors:** Juliana M Haggerty, Xiao N Wang, Anne Dickinson, Chris J O’Malley, Elaine B Martin

**Affiliations:** 1 Biopharmaceutical Bioprocessing Technology Centre, Chemical Engineering and Advanced Materials, Newcastle University, Newcastle-upon-Tyne, UK; 2 Alcyomics Ltd. Framlington Place, Newcastle-upon-Tyne, UK; 3 Institute of Cellular Medicine, Newcastle University, Newcastle-upon-Tyne, UK

**Keywords:** Histopathology, Histopathological damage, Segmentation, Epidermis, Colour space, Mathematical morphology, Classification, Design of experiments

## Abstract

**Background:**

Digital image analysis has the potential to address issues surrounding traditional histological techniques including a lack of objectivity and high variability, through the application of quantitative analysis. A key initial step in image analysis is the identification of regions of interest. A widely applied methodology is that of segmentation. This paper proposes the application of image analysis techniques to segment skin tissue with varying degrees of histopathological damage. The segmentation of human tissue is challenging as a consequence of the complexity of the tissue structures and inconsistencies in tissue preparation, hence there is a need for a new robust method with the capability to handle the additional challenges materialising from histopathological damage.

**Methods:**

A new algorithm has been developed which combines enhanced colour information, created following a transformation to the L*a*b* colourspace, with general image intensity information. A colour normalisation step is included to enhance the algorithm’s robustness to variations in the lighting and staining of the input images. The resulting optimised image is subjected to thresholding and the segmentation is fine-tuned using a combination of morphological processing and object classification rules. The segmentation algorithm was tested on 40 digital images of haematoxylin & eosin (H&E) stained skin biopsies. Accuracy, sensitivity and specificity of the algorithmic procedure were assessed through the comparison of the proposed methodology against manual methods.

**Results:**

Experimental results show the proposed fully automated methodology segments the epidermis with a mean specificity of 97.7%, a mean sensitivity of 89.4% and a mean accuracy of 96.5%. When a simple user interaction step is included, the specificity increases to 98.0%, the sensitivity to 91.0% and the accuracy to 96.8%. The algorithm segments effectively for different severities of tissue damage.

**Conclusions:**

Epidermal segmentation is a crucial first step in a range of applications including melanoma detection and the assessment of histopathological damage in skin. The proposed methodology is able to segment the epidermis with different levels of histological damage. The basic method framework could be applied to segmentation of other epithelial tissues.

## Background

Histopathology refers to the microscopic examination of plant and animal cell tissue for the study and diagnosis of disease through expert medical interpretation. It is used to determine the cause of death through autopsy, in the diagnosis, grading and monitoring of disease, and is the “gold standard” in cancer diagnosis [1]. A small piece of tissue (often from a lesion or tumour) is removed surgically, then chemically fixed, embedded in paraffin, sectioned into thin slices before mounting onto glass slides and staining to enhance the contrast of the tissue structures. The slides are then ready to be graded, which involves the examination of the tissue by an expert, who will look for particular features associated with the disease of interest.

The manual diagnostic methods used in histopathology are time and labour-intensive, and the lack of quantitative characterisation can lead to issues relating to subjectivity and inter and intra-observer variability [2-5]. The use of digital image analysis is becoming widespread due to its potential to address some of these long standing challenges [6].However the application of image analysis in histopathology is challenging due to the high data density of histopathology images, the complexity of the tissue structures, and the inconsistencies in tissue preparation. Recent advances in digital slide scanning, data storage, computational power and algorithm development have resulted in accelerated progress in this field. Digital image analysis can also be used to facilitate research into disease mechanisms, and aid pathologists in terms of detection, diagnosis and classification in both clinical and research settings. Automated techniques applied in histopathology include content based image retrieval [7], image processing [8], segmentation [9,10], feature extraction [11] and classification [12].

### Segmentation in histopathology

This paper is concerned with segmentation, a process in which an image is partitioned into constituent parts or objects, comprising sets of pixels. Segmentation is a critical first step in many image analysis applications since by locating regions of interest early in the analysis subsequent steps can become more accurate and computationally efficient. In histopathology, segmentation is usually used to identify the presence, number, distribution, size and morphology of diagnostic features including tumours, specific cells, nuclei and glands. The accurate identification of these structures is an essential first step in the diagnosis, staging and grading of disease using image analysis. While there have been a number of methods proposed for nuclear and individual cell segmentation in H&E stained tissues and segmentation of structures such as glands [13-15], there are few which attempt to segment particular tissue types as a whole, a useful first step in identifying disease features.

### Challenges of histopathological images for traditional segmentation approaches

A full review of segmentation approaches is beyond the scope of this paper and thorough reviews have been published by Sahoo *et al.*[16] and Segzin and Sankur [17]. A brief discussion of the limitations of traditional segmentation techniques in histopathology follows, but a comprehensive review and discussion of segmentation approaches being used in histology for global scene segmentation and local structure, cell and nuclear segmentation can be found in the review paper by Gurcan *et al.*[6].

Traditionally, segmentation approaches can be sub-divided into contour or edge detection based methods, and region or histogram based approaches. Contour-based approaches identify discontinuities within an image to identify boundaries, based on either simple edge detection or more complex techniques such as active contours [18,19]. However the inherent structural complexity of histopathology images and the frequent presence of overlapping objects make the application of these approaches in histopathology problematic [10]. The skin images used in this research contain a number significant discontinuities identified by edge detection algorithms, including the loose, linear surface layers of the epidermis (the *stratum corneum*), the fibrous structures of connective tissue in the dermis tissue, the basement membrane at the junction of the epidermis and dermis, cleft boundaries at the dermal-epidermal junction and cell membrane boundaries. The large number of potential ‘edges’ in the images make the use of edge or contour based approaches to find the boundary of the epidermis tissue difficult and prone to error.

Region-based methods create sets using pixel or neighbourhood properties such as colour, intensity, location or texture. Location cannot be used for the skin images used in this research, as the orientation and structure of the images varies significantly. Colour, intensity and texture are more applicable as the different tissue types (epidermis and dermis) stain differently and have different morphology (and therefore texture).

Thresholding is the simplest of the region-based methods. It involves selecting an intensity threshold to create a binary image with the two image states representing foreground and background. While the threshold can be selected manually, automating the process is quicker and more objective. To select an appropriate method the particular dataset and problem must be considered. The aims in this research are to threshold the epidermis as foreground and leave the dermis tissue as background. So, a threshold must be chosen to separate the epidermis and dermis pixel sets. The relative proportion of epidermis varies between images and so algorithms based on the percentage of foreground pixels are not useful. Two simple methods for choosing the threshold automatically are the ‘minimum’ algorithm, which smooths the image histogram with until two maxima remain then chooses a threshold *y*_*t*_ such that *y*_*t*_ – 1 > *y*_*t*_ ≤ *y*_*t*_ + 1, and the ‘intermodes’ algorithm which finds local maxima, *y*_*j*_ and *y*_*k*_ and sets the threshold to (*j* + *k*)/2 [20]. However neither of these approaches work well with very unequal peaks, which can be the case for the images used in this research. An alternative approach is the ‘intermeans’ algorithm which iteratively adjusts the threshold so it lies half-way between the means of the background and foreground pixels sets [21,22]. However this technique tends to find a threshold which splits the pixels into two sets of approximately equal number, and this would not be appropriate for the skin images used in this research, which having varying proportions of dermis and epidermis tissue.

One of the most popular approaches for automatic threshold selection, proposed by Otsu in 1979, chooses a threshold to minimise intra-class variance in the foreground and background pixel sets [23]. This approach of minimising variance within each set has potential as there is usually an intensity difference in the pixels in the dermis and epidermis. However, the variation within the epidermis and dermis pixel sets (the inter-class variance) due to differential staining of the different cell and tissue structures means that the method cannot be used on the raw image. In addition to the normal biological variation within a tissue sample and between different patients, there is significant image variation in histopathology images caused by inconsistencies in sample storage, preparation and staining. This variation causes problems when using thresholding techniques, including Otsu’s method, which work best when there is relatively little variation within a set of images [6].

### Approaches to tissue segmentation in histopathology

The use of hybrid segmentation methods is becoming more common as researchers find that a single technique is unable to segment all structures adequately; multi-resolution approaches, feature based classifiers and post-processing steps are all popular additions to the traditional segmentation approaches [9,24]. Some of these hybrid methods used specifically for the segmentation of tissue are described in the following section.

Wang *et al.*[25] described an approach for the segmentation of squamous epithelium from cervical virtual slides using a multi-resolution approach. They used block based texture features in a support vector machine algorithm to create a rough segmentation at x2 magnification, then fine-tuned at x40 magnification. They reported excellent accuracies of 94.9 – 96.3%, however performance statistics were only reported for two of the 20 test images, in addition, sensitivity and specificity were not quoted and it is noted in the paper that the approach tends to misclassify red blood cells and columnar epithelium cells. The algorithm was reported to take 21 minutes to segment one image on a 120000 × 80000 pixel image on a on a Pentium 4 3.4GHz processor with 2GB RAM, which can be scaled to approximately 7,619,048 pixels per second.

Datar *et al.*[26] segmented prostate tissue microarrays into their constituent tissue types, using Hierarchical Self-Organizing Maps to classify pixels based on colour and texture features, followed by unsupervised colour merging. While the segmented images appear to show good performance against a benchmark method, it is not possible to quantitatively compare the results with other methods as no accuracy metrics are quoted and no indication of computational efficiency or segmentation time is given.

Eramian *et al.*[27] presented a graph-cut method to segment epithelium in haematoxylin & eosin (H&E) stained samples of odontogenic cysts. They also included a luminance and chrominance standardization procedure to reduce the variation stemming from sample preparation. For a set of 35 test images they reported mean sensitivity and specificities of 91.5 ± 14% and 85.1 ± 19%respectively, and a mean segmentation accuracy of 85 ± 16%. The average runtime of their method was 7.2 s per image, which can be scaled to 189,583 pixels per second.

In this paper, a three-stage process for the segmentation of histological images is presented. The three-stage process consists of: (1) colour image pre-processing primarily for the purpose of contrast enhancement, (2) Otsu thresholding and (3) morphological processing and object classification of the binary segmentation mask. The developed method is a novel way of enabling highly variable sets of complex histopathological images to be robustly segmented using the simple and easily implemented Otsu thresholding method. Unlike the multi-resolution approach of Wang *et al.*[25] which requires images at x2 and x40 magnification, this procedure can be carried out on a single image at x10 magnification (a relatively low magnification was chosen to reduce memory requirements and processing time).The traditional thresholding approach was improved with pre-processing of the colour image prior to thresholding and post-processing of the binary image produced by the thresholding operation. This is a similar approach to that used by Eramian *et al.*[27], who included a pre-segmentation colour standardisation and post-segmentation processing step based on domain specific rules. The previously published methods for tissue segmentation all use classification or clustering of single pixels or pixel sets based on a feature vector of properties, with support vector machines used by Wang *et al.* Hierarchical Self-organizing Maps used by Datar *et al.*[26]and a graph cut method by Eramian *et al*. The three-stage process presented here enables the use of a traditional and well understood thresholding technique in a challenging domain in which it would not ordinarily give good results.

The process has been optimised and tested on whole slide images of H&E stained human skin sections that exhibited varying levels of histopathological damage including vacuolisation, sub-epidermal cleft formation, dyskeratosis and necrosis. The specific damage in these images is located in the epidermis, and so this paper will focus specifically on the use of the algorithm to segment epidermis tissue in complex skin images. The process framework can be optimised to segment particular morphological features in addition to whole tissue. The presence of varying levels of structural damage and image variation created by inconsistencies in tissue preparation and staining makes accurate segmentation of specific tissue types particularly challenging for this dataset. The work forms part of a research project to automate the histological grading of an immunological tissue response in a human skin explant assay [28]. The development of a segmentation algorithm able to handle the challenges of this dataset is critical to the creation of a fully automated computer assisted process for histological grading in such an assay.

### Mathematical morphology

Mathematical morphological operations are particularly useful for object recognition in image analysis, as the operations can preserve the key shape characteristics of an image while removing uninformative variations in intensity [29]. First described by Georges Matheron [30], mathematical morphology is a theoretical approach to the analysis of geometric structures and encompasses a range of operations utilising set theory.

**Figure 1:**
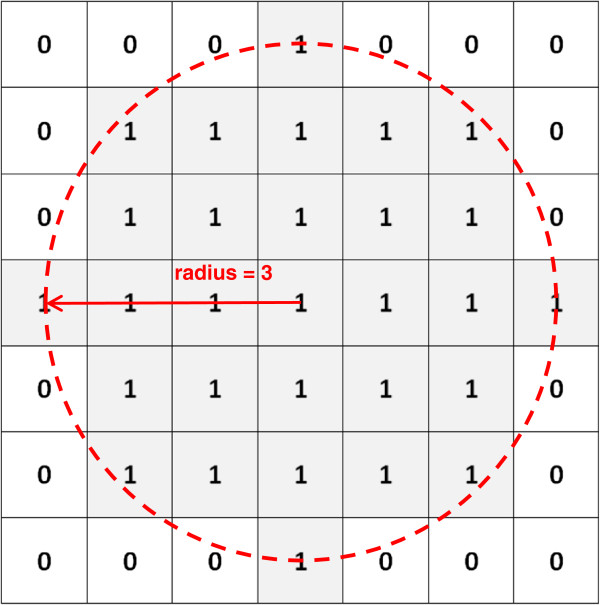
**Example of a disk shaped structuring element.** Figure 1 shows the matrix which defines a disk shaped structuring element with a radius of 3. The location of the 1 s in the matrix defines the neighbourhood for the morphological operation.

Morphological processing operations require the interaction of an input image pixel set with an external pixel set in the form of a structuring element (SE). A SE is a 2 or 3 dimensional matrix of 0’s and 1’s, generally much smaller than the image being processed, in which the 1’s define the shape and size of a pixel neighbourhood; a disk shaped SE of radius 3 is shown in Figure 1. Shapes within the input image are analysed using a suitably shaped SE, and the output image pixels are based on a comparison of the corresponding pixel in the input image with its neighbourhood, as defined by the SE. By varying the size and shape of the SE, it is possible to extract shape information for different parts of the image.

Dilation and erosion are the operators most commonly used in binary mathematical morphology, and are the basis of most other operations. Dilation tends to make objects bigger, smooth uneven edges and bridge gaps, while erosion tends to make objects smaller, remove protuberances and break bridges. In *dilation*, pixels are added to the boundaries of objects using a rule stating that the output pixel is set to 1 if any pixels in the input neighbourhood are 1. If *A* is a set of pixels in the input image and *B* is a structuring element, (*B*’)_s_ is a reflection of *B* about its origin, followed by a shift by s. Dilation, which is denoted by A⊕B is equal to:

$$A⊕B=\left\{\left(a\right|{\left(B'\right)}_{s}\cap A\neq \emptyset \right\}$$

*Erosion* refers to the combination of the SE and image sets by vector subtraction consequently pixels are removed from the outside of objects based on a rule stating that the output pixel is set to 0 if any pixels in the input neighbourhood are 0. The erosion of set *A*, by structuring element, *B* is given by:

$$A⊖B=\left\{\left(a\right|{\left(B\right)}_{a}\subset A\right\}$$

### Colourspaces

The ***RGB*** system is one approach to representing colour, however the perception of colour can be specified, created and visualised using a number of other representations termed colourspaces. Colourspaces are mathematical models which represent colour using colour components; they can exist in 2D, 3D or 4D, thus a set of 2, 3 or 4 coordinates can be used to express any colour and its position within the colourspace. Many colourspaces attempt to separate the lightness from the colour components as this is a significant weakness in the ***RGB*** colour space.

## Methods

**Figure 2:**
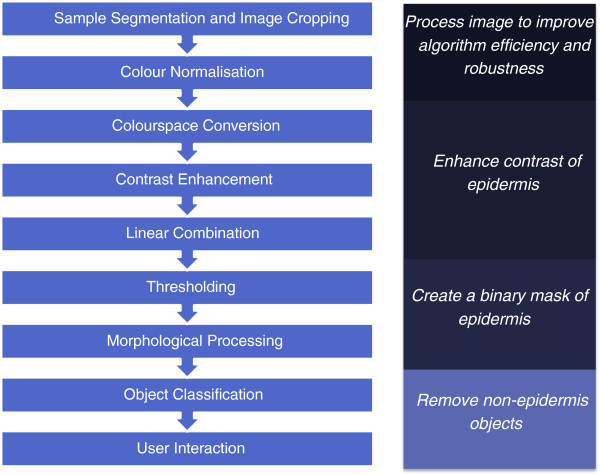
**Summary of stages in the developed epidermis segmentation algorithm.** Figure 2 summarizes the main stages in the algorithm to segment the epidermis from a digital image of an H&E stained skin section. The initial steps of sample segmentation, image cropping and colour normalization process the image so that subsequent steps are more efficient and robust. The colourspace conversion, contrast enhancement and linear combination steps enhance the contrast between the epidermis and the rest of the tissue; this makes the thresholding step that follows more effective. Morphological processing fine tunes the segmentation of the binary image created by thresholding. The object classification step removes objects in the image which do not satisfy criteria set out for objects that are part of the epidermis, the user interaction step allows a human operator to change the classification of objects performed in the previous step.

In this section, the skin explant assay used to generate the skin samples is described, followed by the image acquisition procedure and the methodology used to pre-process, threshold and fine-tune the segmentation of the epidermis tissue. Figure 2 summarises the main stages of the segmentation algorithm. The algorithm is based on the differences in the colour and intensity of staining in the different tissue types, the texture within each tissue and the overall shape and size of tissue regions. A colour normalisation step is also included to handle variations in the chromacity of the stained tissue caused by differences in sample thickness, staining procedure, and lighting during sample preparation and image acquisition. The algorithm was implemented using the Image Processing Toolbox™ in MATLAB®, Version 7.11, R2010b (The MathWorks, Inc.).

### Data source: skin explant assay

The skin samples forming the test set were generated in a skin explant assay [31] developed to predict and investigate the immunobiology of graft versus host disease occurring post hematopoietic stem cell transplantation in HLA-matched siblings. Punch skin biopsies of 4 mm diameter were taken from the back below the waist-line of transplant patients after informed consent. Each 4 mm^2^ skin biopsy was equally dissected into 4 small sections and co-cultured with pre-primed alloreactive T cells. Skin sections cultured in medium alone were used as controls. After 72 hr incubation at 37°C, skin sections were fixed in formalin, sectioned and stained with H&E. The manual histopathological grading was assessed and confirmed independently by two experts. The histopathological grading system was as follows: grade I, mild vacuolisation of epidermal basal cells; grade II, diffuse vacuolisation of basal cells with scattered dyskeratotic bodies; grade III, subepidermal cleft formation; grade IV, complete epidermal separation. The assay is described fully in Sviland and Dickinson [31]. In addition to the vacuolisation, cleft formation and the presence of dyskeratotic bodies, some of the images also included regions of necrotic tissue. Ordinarily, samples with necrotic tissue are not manually scored and biopsies with such artefacts are not included in the standard assay readout, however these images were included here to enable the software to identify and ignore artefacts or necrotic regions. In the 40 sample data set of skin explant slides, there were eleven grade I examples, twelve grade II, nine grade III and eight grade IV.

### Ethics statement

All skin samples were obtained from patients after informed consent. All human material was collected and stored using protocols approved by Local Research Ethics Committees (Newcastle and North Tyneside Hospitals NHS Trust).

### Image acquisition

**Figure 3:**
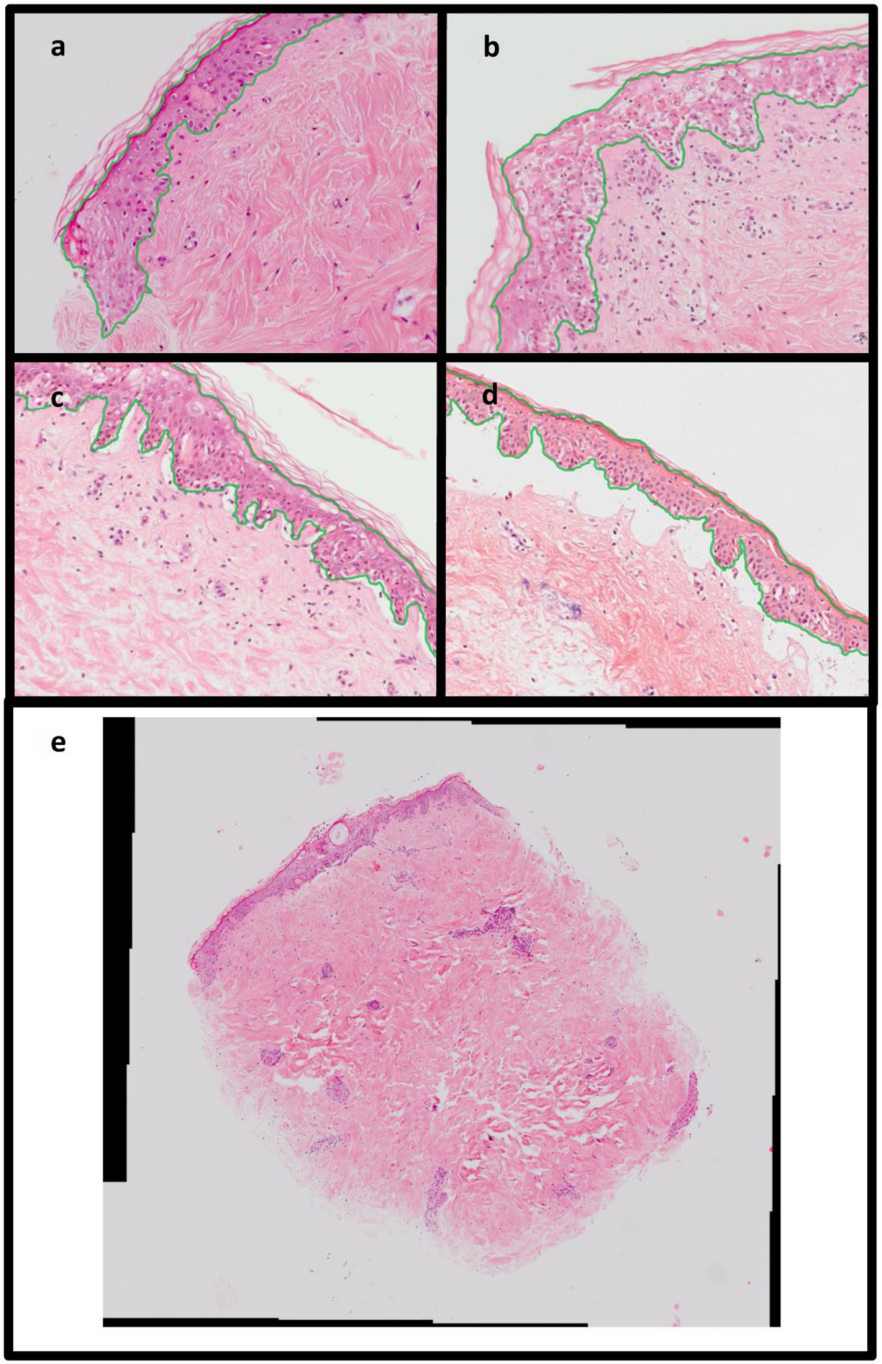
**Digital images of H&E stained skin showing varying levels of histological damage.** Figure 3 shows examples of H&E stained skin sections with each grade of damage, with grade I showing a slight vacuolisation of epidermal basal cells (Figure 3**a**), grade II more diffuse vacuolisation plus dyskeratotic bodies (Figure 3**b**), grade III showing sub-epidermal cleft formation in addition to grade II changes (Figure 3**c**), and finally grade IV showing complete epidermal separation (Figure 3**d**). The epidermis tissue to be segmented is outlined in green in each image. Figure 3**e** is a typical tiled image generated by the Zeiss AxioImager II system, the black regions around the edges are added to form a rectangle after the image tiling and stitching.

Digital images of the sectioned and stained skin samples were created using a Zeiss AxioImager II system with MOSAIC tiling facility. The image tiling and stitching facilities were used to create single x10 magnification RGB images of each skin sample. The image dimensions and aspect ratios varied, with heights of 1047 to 4819 pixels, and widths from 2676 to 5254 pixels. Figure 3 shows examples of stained skin sections with damage grades I-IV (Figure 3a-d) with the epidermis tissue outlined in green, and an example of a typical whole slide image generated by the Zeiss AxioImager system (Figure 3e).

The remainder of the methods section is used to describe the eight individual steps in the epidermis segmentation algorithm in detail.

### Sample segmentation and image cropping

The first stage in the algorithm is to segment the pixels in the image representing the skin sample. This first segmentation increases the efficiency of the algorithm by limiting the number of pixels being processed during subsequent steps. While segmentation of the skin sample could be achieved by locating either the background or the sample pixels, the background pixels are used as they have lower inter and intra-image variance. The background pixels are located by creating a composite image, ***K***, by summing the red, green and blue (***R***, ***G*** and ***B***) intensities for each pixel in the ***RGB*** image, then taking the most frequently occurring value in *K* to approximate the background colour and storing this value as the background threshold, *bg*_*thresh*_. Black pixels present at the image edges due to the image tiling procedure are excluded by determining the mode of non-zero elements in ***K***.

$$\begin{array}{c} K=R+G+B\\ bg_{\mathrm{thresh}}=\mathrm{mode}\;\left(\left\{\left(k_{\mathrm{ij}}\right|k_{\mathrm{ij}}\;\mathrm{is}\;\mathrm{an}\;\mathrm{element}\;\mathrm{of}\;K\;\mathrm{and}\;k_{\mathrm{ij}}>0\right\}\right) \end{array}$$

**Figure 4:**
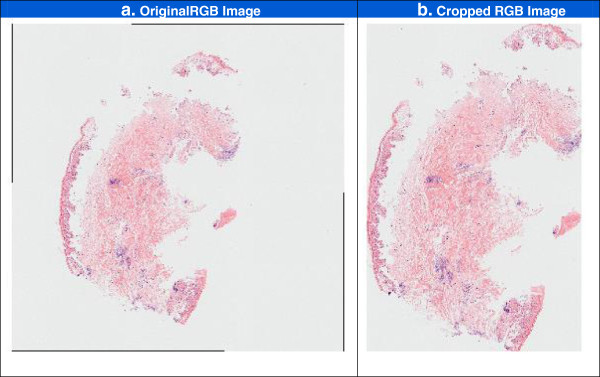
**Example of image cropping on RGB images.** Figure 4 shows the effect of the automated image cropping procedure on an image which includes a number of small tissue fragments. The original image is shown in Figure 4**a**, and the cropped image is shown in Figure 4**b**.

The black pixels at the image edge (as shown in Figure 3e) are replaced with the background threshold intensity, *bg*_*thresh*_, to create a consistent background. To reduce memory requirements in the implementation of this algorithm, excess outer rows and columns of background pixels which do not intersect the sample are cropped at this stage. For an *m x n* size image, this is done by cropping any rows where the sum of composite pixels in the row is less than *bg*_thresh_*n (an average value for a background row), and cropping any columns where the sum of composite pixels in the column is less than *bg*_thresh_*m. The process of cropping is shown in Figure 4 for a particularly challenging image showing grade IV damage. The original image is shown in Figure 4a, and the in Figure 4b all excess background has been cropped without cropping more than a few pixels of tissue at the sample edges.

Prior to thresholding the colour image is smoothed using an averaging filter which replaces each image pixel with the mean value of its pixel neighbours in each colour channel. This has the effect of reducing variation within the background pixel and the sample pixel sets and facilitating the choice of threshold when creating a binary sample mask. The operation is performed using convolution with a kernel filter to represent the pixel neighbourhood (***K***_*smoothed*_ = ***K*** * kernel). Initially a filter size of 19×19 (where each element is 1/(19*19)) was chosen based on the typical size of vacuoles and clefts in the image which needed to be smoothed.

A new binary image *sampleMask* is created by thresholding the smoothed image, ***K***_*smoothed*_. The value of the threshold was based on *bg*_*thresh*_ , however since *bg*_*thresh*_ is a measure of central tendency, the actual threshold used must be lower to ensure the majority of background pixels are below it. To calculate how much lower the threshold needed to be, the standard deviation of the background pixels in all 40 smoothed composite images was found. The standard deviation ranged between 1.2 and 3.2, subtracting three standard deviations of the image with the highest variance (3*3.2) from *bg*_*thresh*_ will mean the threshold will fall below the intensity of the majority of background pixels.

$$\mathrm{sampleMas}k_{i,j}=\left\{\begin{array}{c} 1\;\mathrm{if}\;K_{i.j}>bg_{\mathrm{thresh}}–9.6\;\\ \;0\;\mathrm{if}\;K_{i.j}\leq bg_{\mathrm{thresh}}–9.6 \end{array}\right)$$

**Figure 5:**
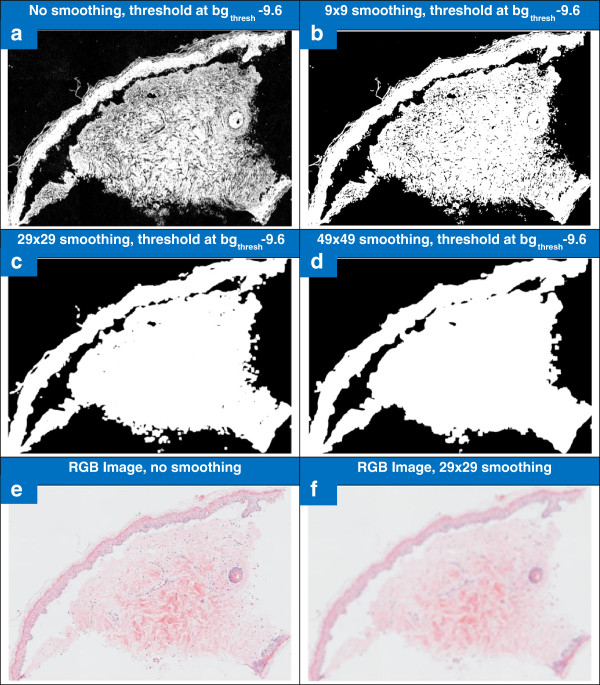
**Effect of mean filtering and mean filter sizing on RGB image and thresholding.** Figure 5**a**-**d** show the addition of a pre thresholding smoothing step affects the subsequent thresholding operation. Figure 5**a** shows the binary mask created by the thresholding operation without smoothing, while Figure 5**b**, **c** and **d** show the binary mask when the smoothing step is done using a 9×9, 29×29 and 49×49 sized filter. Figure 5**e** shows an RGB image and 5**f** shows the same image once it has been smoothed with a 29×29 filter, the filter has the effect of blurring minor variation within the tissue types.

The effect of the averaging filter and its size on the thresholding operation is shown in Figure 5. A variety of kernel filter sizes were tested to determine their effect on the thresholding step. The post thresholding binary mask created after pre-processing with no smoothing is shown in Figure 5a, with a 9×9 mean filter kernel in Figure 5b, a 29×29 filter in Figure 5c, and a 49×49 filter in Figure 5d. The dimensions of the mean filter must be large enough to smooth the coarse texture in the lower parts of the dermis and clefts at the dermal-epidermal junction so these features are included in the *sampleMask*, while minimising loss of accuracy at the perimeter of the sample. Without any smoothing the thresholding results in a mask which is very detailed but does not include any vacuoles or clefts. The smoothing creates a simpler mask which includes gradually more of clefts, vacuoles and white regions within the dermis as the filter size is increased. While mean filters of sizes between 9 and 49 can be used successfully, an intermediate value of 29 was chosen for two main reasons. Firstly, at this size the thresholded mask tends to have fewer separate objects than when smaller filters are used meaning a hole filling operation can be used subsequently to create a simple mask including clefts and vacuoles. Secondly, the 29×29 filter size was chosen in preference to a larger filter as the larger filter could result in a loss of segmentation accuracy at the sample boundary. The original RGB image and the image after smoothing with the 29×29 filter are shown in Figure 5e and f; there is a strong blurring effect which removes detail from the internal parts of the tissue.

In some images the dermal epidermal clefts are very large the smoothing operation is not enough to include them in the sample mask as foreground objects in the *sampleMask* (as seen in Figure 5). Mathematical morphology is used to in-fill these “holes” in the binary sample mask and also to remove small objects such as dust or tissue debris on the slide which have been captured during thresholding, but which are not useful for subsequent analysis. The sequence of operations used to refine the segmentation is as follows.

Step 1: Fill holes - Fills internal regions of background pixels within foreground objects in the binary image using the MATLAB, imfill function, an implementation of morphological reconstruction described in [32].

Step 2: Remove small objects - Removes foreground objects that consist of less than 25,000 connected pixels. This value was chosen so that the smallest fragments of tissue found in the image set used in this research were not excluded, but the value was high enough to exclude smaller objects such as dust or other tissue debris.

### Colour normalisation

The initial optimisation of the method and parameters was carried out without colour normalisation, however the addition of this step improves the performance in terms of sensitivity, specificity and overall accuracy. The relative improvement is described in the results section. Staining inconsistencies in the input images are addressed by mapping the histogram for each individual colour channel of the RGB image to those of a target image, *I*_*ref*_, identified as well stained by an expert histopathologist. Only the sample pixels identified in the previous sample segmentation step are included in this colour normalisation.

The best approximation of the target image is obtained through the application of a grayscale transformation, *T*, to the sample pixel intensities, *k*, in the input image. A transform is selected for each colour plane so as to minimise the difference between the cumulative histogram of the transformed input image intensities, *c*_*input*_ and the cumulative histogram of the well stained target histogram, *c*_*ref*_. The function to be minimised is

$$\;\left|c_{\mathrm{input}\;}\left(T\left(K\right)\right)-c_{\mathrm{ref}}\left(k\right)\right|$$

which can be implemented in MATLAB using the function *histeq*[26].

**Figure 6:**
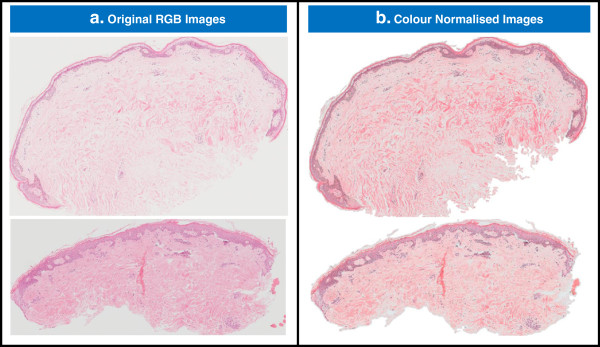
**The effect of colour normalisation on RGB skin images.** Figure 6**a** shows two RGB skin images with different staining contrast, slightly different lighting during acquisition, different overall colour hues, and different proportions of epidermis and dermis tissue. Figure 6**b** shows the same images once they have undergone colour normalisation. The non-sample pixels have been changed to white in the normalised images.

The effect of the colour normalisation on two images with significant differences in staining and lighting is shown in Figure 6. The original images are shown in Figure 6a and in Figure 6b the non-sample pixels have been changed to white and the sample pixels have been normalised. The final two images have a similar contrast between the epidermis and dermis, and a similar range of colour hues and saturation to each other.

### Colourspace conversion

The next part of the segmentation procedure is a coarse segmentation of the epidermis based on the thresholding of a high contrast composite image. The composite image is created using a weighted linear combination of contrast stretched images in colour spaces which enhance the contrast between the regions of interest and the rest of the image.

A number of colour spaces were investigated to identify a representation that would maximise the contrast between the epidermis and the rest of the skin tissue. Those tested included ***CMYK*** (cyan, magenta, yellow and black) which is based on subtractive colour mixing, ***HSV*** (hue, saturation and value), ***YCbCr*** (luminance, blue chrominance and red chrominance) and the L*a*b* (lightness, red/green, yellow/blue) colour space [27].

The ***Cb*** channel in the ***YCbCr*** colour space and the b* channel in the L*a*b* colourspace were observed to provide good contrast between the epidermis and the rest of the tissue. The ***Cb*** (blue chrominance) and the b* (yellow/blue) colour channels both highlight the blue staining of haematoxylin which stains the nuclei in the cells of the epidermis. Although there are nuclei-containing cells present in the dermis, they are few in number.

When the two colourspaces were tested on a set of 16 images chosen to include different damage levels and staining variation, with optimised contrast enhancement, the b* image enhanced the contrast of the tissue types more consistently than the ***Cb*** plane. The b* plane of the L*a*b* colourspace was therefore chosen for use in the algorithm. The CIE L*a*b* specified by the International Commission of Illumination in 1976, is a perceptually uniform colourspace based around human perception of colour. In the CIE L*a*b*, colour is represented on three axes: (1) lightness, with the maximum value a perfect reflecting diffuser and the minimum representing black, (2) a red to green axis, with a positive a* value showing red, and a negative a* showing green, and (3) a yellow to blue axis, with a positive b* value showing yellow, and a negative b* showing blue.

It was noted that during optimisation of the subsequent contrast enhancement and thresholding stages that when the ***RGB*** image was converted to grayscale and contrast enhanced, good contrast for the epidermis was observed in some images where the b* colour channel was displaying poor contrast. The images showing poor contrast of the epidermis in the b* colour channel tended to have weak nuclear staining by haemotoxylin, which appears as a strong blue/purple colour and therefore stands out in this yellow/ blue colour channel. The complement image of the grayscale representation highlights more intensely stained areas, despite not being as specific to particular colours as the b* plane and tends to highlight both the cytoplasm and nuclei in the epidermis which are usually stained more intensely than the dermis tissue. When tested on a set of 25 images, the image variation meant that in different images the epidermis was highlighted best in either the b* or the grayscale image, it was therefore decided that a combination of the data in the grayscale and b* images could be used to enhance the robustness of the following steps.

### Contrast enhancement

Contrast enhancement by stretching transforms a low contrast image, b*, to a high contrast image, ***b’***, by remapping the gray levels to cover a wider dynamic range. A linear transformation maps the lowest gray level in b*, ***GL***_***min***_, to a new minimum gray level ***GL’***_***min***_, and the highest gray level in b*, ***GL***_***max***_, to a new maximum gray level ***GL’***_***max***_. The linear transformation that preserves the intensity histogram shape is given by:

$${b^{'}}_{i,j}=\mathrm{INT}\left\{\frac{G{L^{'}}_{\max}-G{L^{'}}_{\min}}{GL_{\max}-GL_{\min}}\left[{b^{*}}_{i,j}-GL_{\min}\right]+G{L^{'}}_{\min}\right\}$$

where ***INT*** returns an integer value. ***GL***_***min***_ and ***GL***_***max***_ can be replaced with the penetration points ***P***_***min***_ and ***P***_***max***_, to remap a band of intensities. Penetration points can be determined using a cumulative percentage histogram; for example, selecting the intensities to exclude the top and bottom 1% of the data. This is a useful technique if the intensity band of interest is at a mid-gray level rather than at an extreme gray level.

Only sample pixels were included in the contrast enhancement process, as the aim was to maximise the contrast between the dermis and epidermis and background pixels are not relevant to the rest of the process. Remapping a narrow, more specific band of intensities was investigated to try and improve the contrast. When tested manually using a variety of absolute intensity levels as penetration points, the optimal intensity band for remapping to enhance contrast of the epidermis varied significantly for different images. This issue was addressed by determining penetration points based on the cumulative percentage histogram so that a set percentage of low and high intensity pixels were saturated in the final image. This method is more able to cope with any remaining staining variation and pixel intensity outliers than choosing absolute intensity levels. This approach was used on both the b* and grayscale image planes to create new contrast enhanced images, ***b’*** and ***G’***.

Following the contrast enhancement, the two images ***G’*** and ***b’*** were smoothed using an averaging mean filter, as described for sample segmentation. The operation is performed using convolution with a kernel filter to represent the pixel neighbourhood (***K***_***smoothed***_ = ***K*** * kernel). This has the effect of reducing variation within the sample pixels and smoothing minor variations within the epidermis and dermis regions and leaving the main variations due to differences in the two tissue types. This will facilitate the choice of threshold in method step 6.

As the contrast enhancement and smoothing are critical steps in the process, the optimal values for the upper and lower penetration points for the grayscale and b* images were determined along with the optimal sizes of smoothing mean filter and the structuring element used in the morphological processing (method step 7). This optimisation varied the 6 key parameters within defined ranges using a Design of Experiments approach. A quadratic model was then built and used to select parameter values which maximised sensitivity and specificity. The process and results are described in detail in the results section.

Based on the optimisation the 29.1% of pixels with lowest intensity in the grayscale image ***G’*** were saturated and the remaining pixels remapped to utilise the full dynamic range. For the red-green ***b’*** image, the 36.7% of pixels with the lowest intensity were saturated and the remaining pixels remapped to utilise the full dynamic range. A mean filter size of 41×41 (where each element is 1/(41*41)) was chosen during the optimisation.

### Linear combination

An equal weighted linear combination is then applied to the two enhanced and smoothed images ***G’*** and ***b’*** to create a new image, ***Gb***:

$$Gb_{i,j}=\;0.5\times G{'}_{\mathrm{ij}}+\;0.5\times b{'}_{\mathrm{ij}}$$

Combining the two images captures both staining intensity and staining colour information in a single grayscale image.

### Thresholding

Following colour normalisation and contrast enhancement, Otsu’s automated method [23] was applied to determine the optimal threshold based on the intensity distribution of sample pixels in the combined, enhanced image, ***GB’***. The Otsu method uses discriminant analysis to determine a threshold, ***Level***, which maximises the separability of two pixel classes by minimising the intra-class variance. In this process, the aim is for this method to maximise separability of dermis and epidermis pixels. ***GB’*** is converted into a binary image, ***BW***, using the threshold, ***Level***. Any non-sample pixels are changed to black (as background).

$$BW_{i,j}=\left\{\begin{array}{c} 1\;\mathrm{if}\;\mathrm{GB}{'}_{i.j}>\mathrm{Level}\\ 0\;\mathrm{if}\;\mathrm{GB}{'}_{i.j}\leq \mathrm{Level} \end{array}\right)\;$$

**Figure 7:**
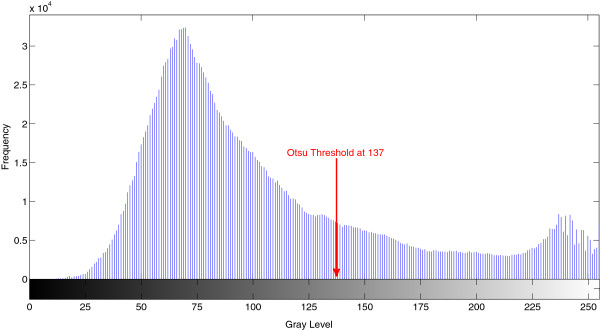
**Histogram of enhanced additive image showing Otsu threshold.** Figure 7 shows the intensity histogram of image pre-thresholding. The image has been created by combining a contrast enhanced b* image plane and a contrast enhanced grayscale image. The optimal threshold calculated using Otsu’s method is labelled on the histogram.

Figure 7 shows a histogram of a typical ***GB’*** image. The method assumes an approximately bimodal distribution. The Otsu threshold, ***Level***, generated using Otsu method is labelled in the figure at the intersection of the upper and lower intensity components.

### Morphological processing

Morphological processing is used to further process the binary image, by removing small misclassified objects, merging multiple objects, in-filling holes and closing gaps. These operations are applied to the whole image, however once the operations are completed, any non-sample pixels which may have been affected are changed back to black. The sequence of operations summarised below is used to refine the segmentation. The choice of structuring element size (radius = 8) for the morphological closing and opening steps was optimised based on the final sensitivity and specificity of the algorithm as described in the results Section. A disk shaped structuring element was used as this shape reflects biological structures more accurately than sharp angles or linear shapes.

Step 1: Morphological closing - Morphological closing (dilation then erosion) enlarges the boundaries of foreground (bright) objects in the image and closes gaps between them, and shrinks background-coloured holes in the foreground objects. A disk shaped structuring element with *radius = 8 pixels* is employed.

Step 2: Morphological opening - Morphological opening (erosion then dilation) removes some of the foreground (bright) pixels from the edges of foreground objects, which will break fine bridges between objects while preserving the object size. A disk shaped structuring element with *radius = 8 pixels* is employed.

Steps 1 and 2 combine to smooth and simplify the objects edges without changing the size of objects. Simple object perimeters are useful in the image classification algorithm for which this segmentation algorithm is being developed; however these steps could be omitted if this is not an important output of the segmentation.

Step 3: Remove small objects - Remove foreground objects that consist of less than 4000 connected pixels. The threshold of 4000 pixels was decided based on a size assessment of any regions of epidermis identified as epidermis objects at this stage of the process. The majority of epidermis regions at this stage consist of more than 4000 pixels.

Step 4: Fill holes - Fills internal regions of background pixels within foreground objects in the binary image that consist of less than 7000 connected pixels. A threshold is required as in some images there are regions of dermis tissue surrounded by epidermis tissue (due to tissue slicing technique) and if these regions are filled the specificity of the final algorithm is compromised. Again the value was chosen based on the typical size of enclosed dermis regions and other structures within the epidermis which should not be included.

### Object classification

Following morphological processing, the binary mask includes objects that are not part of the epidermis. These include collections of cells within the dermis, which have a similar appearance to epidermis tissue, and parts of the dead surface layer, the *stratum corneum*, which may be segmented with the epidermal tissue in cases where it is highly stained. For each object, *Z*, the object area, *Z*_*Area*_, and the area of the object’s bounding box, *Z*_*BoundBox*_, are determined. The ratio of *Z*_*Area*_ to *Z*_*BoundBox*_ gives the extent, *Z*_*Extent*_, of the object, which is a useful measure for differentiating between the long thin objects of the epidermis and the more compact, circular clusters of cells within the dermis:

$$Z_{\mathrm{Extent}}=\frac{Z_{\mathrm{Area}}}{Z_{\mathrm{BoundBox}}}$$

The *Z*_*Area*_ and *Z*_*Extent*_ can be used to classify the remaining objects as either epidermis or non-epidermis. The thresholded objects are either retained or removed based on their area and their extent and so the effect of changing the area and extent thresholds used in this rule on algorithm sensitivity and specificity was tested. Only objects with an area greater than the area threshold and an extent less than the extent threshold were retained. The values used in this classification were optimised together once all the other critical factors had been set at optimal values, the optimisation is described in the results section. Based on the optimisation the following classification rules were used to classify each object pixel, *z*, in the binary mask.

$$z=\left\{\begin{array}{l} 1\;\left\{\left(\forall z\in Z\right|\;Z_{\mathrm{Extent}}>0.44,Z_{\mathrm{Area}}<20000\right\}\\ 0\;\mathrm{else}\; \end{array}\right)\;$$

where *z* are pixel elements in the object *Z*.

### User interaction

The object classification step can be used to tune the specificity and sensitivity of the final algorithm, however the algorithm also includes the option for the user to interactively include or remove objects for the final epidermis mask. Epidermis segmentation is critical to the performance of the subsequent steps in the skin damage classification that is the ultimate aim of this research, and so this step is available to improve the performance of the algorithm. It is relatively straightforward for a user to determine whether a given object is part of the epidermis when shown next to an image of the RGB image. The user has the option to (1) approve the object selection, (2) click on objects to remove them, or (3) select additional objects, which were removed during object classification.

## Results

The algorithm was evaluated using 40H&E stained skin sections generated in a skin explant assay. The performance of the method was evaluated using a “ground truth” image that was created through the manual mark-up of the epidermis. This was achieved by drawing the boundary of the epidermis onto the original ***RGB*** images in green with the aid of a graphics tablet. The high contrast boundary was easily identified using a thresholding procedure on the red channel of the RGB image. The outlined regions were flood-filled with a morphological reconstruction algorithm implemented using the MATLAB function, *imfill*. The *stratum corneum,* the epidermal surface layer which appears as a looser collection of flaky layers, consists of dead cells that do not provide useful information about the state of damage in the tissue and hence was not included in the manual epidermis mark-up. Evaluation of the segmentation procedure was undertaken by comparing the area of the algorithm-segmented epidermis with the “true” epidermis area generated during manual segmentation.

### Performance metrics

The total number of pixels identified as part of the epidermis in the manual and automated segmentation, and the total number of pixels in each image were used to determine the true positive, true negative, false positive and false negative fractions. The total pixel number in the image was based on the cropped image, to avoid an excessive number of background pixels skewing the results. If *A*_*s*_ and *A*_*t*_ represent the pixel sets identified as epidermis by the algorithm and manual methods respectively, the various fractions can be calculated as follows:

$$\mathrm{False}\;\mathrm{Negative}\;\left(\mathrm{FN}\right)=\left(\mathrm{ImageArea}-A_{s}\right)\cap A_{t}$$

$$\mathrm{False}\;\mathrm{Positive}\;\left(\mathrm{FP}\right)=A_{s}\cap \left(\mathrm{ImageArea}-A_{t}\right)$$

$$\mathrm{True}\;\mathrm{Positive}\;\left(\mathrm{TP}\right)=A_{s}\cap A_{t}$$

$$\mathrm{True}\;\mathrm{Negative}\;\left(\mathrm{FN}\right)=\left(\mathrm{ImageArea}-A_{s}\right)\cap \left(\mathrm{ImageArea}-A_{t}\right)$$

These fractions were used as the basis to calculate the percentage sensitivity, specificity and accuracy of the automated segmentation for each image by comparing the algorithmic method to manual segmentation. The three metrics were calculated as follows:

$$\mathrm{Sensitivity}=\frac{\mathrm{TP}}{\left(\mathrm{TP}+\mathrm{FN}\right)}\times 100$$

$$\mathrm{Specificity}=\frac{\mathrm{TN}}{\left(\mathrm{TN}+\mathrm{FP}\right)}\times 100$$

$$\mathrm{Overall}\;\mathrm{Accuracy}=\frac{\left(\mathrm{TP}+\mathrm{TN}\right)}{\left(\mathrm{TP}+\mathrm{FP}+\mathrm{FN}+\mathrm{TN}\right)}\times 100$$

In combination, the three metrics provide an indication of the performance of the segmentation algorithm. Sensitivity is a measure of how well the algorithm identifies epidermis pixels, while specificity shows how well it identifies non-epidermis pixels. In any detection test, a balance between sensitivity and specificity is required since typically an increase in one will lead to a decrease in the other. The accuracy measurement combines the two metrics within one measurement, and quantifies the percentage of pixels correctly classified as epidermis and non-epidermis when compared to manual segmentation.

### Optimisation of algorithm parameters

A number of key parameters in the algorithm were optimised by running the algorithm without user interaction and assessing the effect of the key parameters on the mean sensitivity and specificity. The algorithm was optimised using 25H&E stained skin sections generated in a skin explant assay. This left 15 skin images as a final test set to check whether the algorithm had been ‘overtrained’ on the test set. The optimisation of the six critical parameters was carried out using a Design of Experiments (DOE) approach using the software program MINITAB v16.2.4. This approach was used so that interaction between the various parameters could be tested and analysed. Initially a 2-level Fractional Factorial design was used to optimise the six parameters (factors) and test their interaction at two levels. The optimisation was run using a Resolution IV design, which involves running ¼ of the possible parameter combinations, with 1 centre point with the factors at intermediate levels. This allowed the analysis to be carried out by running the algorithm 17 times on the 25 image dataset, rather than 64 times, which would be required for a full factorial experiment at two levels. In a Resolution IV design, some effects are confounded, and cannot be estimated separately; however main effects of single factors are unconfounded by two-factor interactions.

In this initial screening run, the values of the parameters and the mean sensitivity and specificity of each run are shown in Table 1. The range of values to test was determined by varying the values manually on 16 images in parallel, and assessing the outcome visually. For instance, for the ***b’*** image values were chosen to accentuate the blue/ purple pixels of the keratinocyte cell nuclei within the epidermis, while for the ***G’*** image values were chosen to highlight the whole of the epidermis including the cytoplasm and cell membranes. A similar manual process was carried out to determine suitable ranges for the smoothing and structuring element sizing.

**Table 1:** Parameter values and responses (mean sensitivity and specificity) for fractional factorial screening test

**Run**	**Gray low**	**Gray high**	b* Low	b* High	**Smooth**	**SE**	**Mean sensitivity**	**Mean specificity**
1	0.2	0.875	0.225	0.95	30	12.5	61.51	96.43
*2	0.3	1	0.3	0.9	40	5	76.88	97.09
3	0.1	1	0.15	0.9	40	20	58.49	97.56
4	0.3	0.75	0.15	0.9	40	5	61.45	93.77
5	0.1	0.75	0.15	1	20	20	20.56	98.29
6	0.3	1	0.15	0.9	20	20	62.59	97.57
7	0.3	0.75	0.15	1	40	20	54.42	94.91
8	0.1	0.75	0.15	0.9	20	5	43.82	95.20
9	0.3	0.75	0.3	1	20	5	62.84	93.68
10	0.1	0.75	0.3	0.9	40	20	35.70	96.59
*11	0.1	1	0.15	1	40	5	72.43	97.03
12	0.3	0.75	0.3	0.9	20	20	41.01	94.48
13	0.1	0.75	0.3	1	40	5	46.89	94.76
14	0.1	1	0.3	0.9	20	5	70.36	96.90
*15	0.3	1	0.15	1	20	5	74.25	97.77
*16	0.3	1	0.3	1	40	20	73.85	97.59
17	0.1	1	0.3	1	20	20	52.65	96.07

The best performing sets of parameters were in runs 2, 11, 15 and 16, which all had sensitivity > 73% and a specificity > 97%. These lines are starred in the first column of Table 1. When the effects of each of the parameters were analysed, the upper threshold of the grayscale image was found to be the most important effect; all the well performing runs had this threshold set at the higher level of 1. The other parameters did not have such clear effects.

A more detailed optimisation was run, fixing the upper gray threshold at 1, but varying the other parameters using a Response Surface Design. This second optimisation looked at more possible levels and combinations, including some extreme points to investigate the relationship of the factors, including possible interactions and curvature in the data. A Central Composite design was used, which includes axial points to investigate extreme conditions and centre points which together enable curvature and second order responses to be investigated.

**Figure 8:**
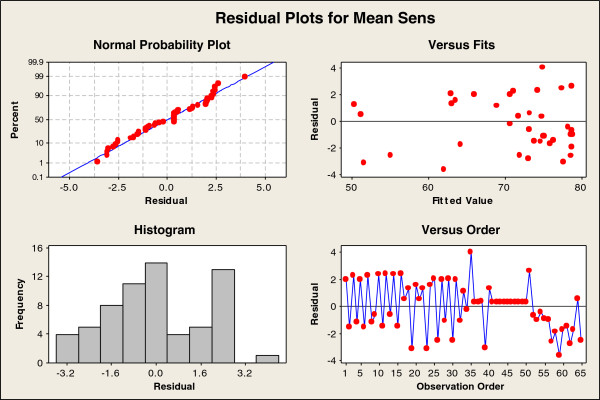
**Residuals plots for quadratic model of key parameter effect on sensitivity.** Figure 8 includes a normal probability plot of the residuals for the predictive model, a histogram of the residuals, a plot of residuals versus fitted value and a plot of residuals vs run order.

The initial 2-Level Factorial data was added to the Response Surface Design before the results were analysed. A full quadratic response surface model was fitted to the data using MINTAB and then insignificant terms (with p-values > 0.05 in the Analysis of Variance table) were removed one at a time until all remaining terms had p-values of < 0.05. The high b* threshold was not a significant term in the model so it was removed along with any squared and interaction terms it was included in. The remaining terms in the final model along with the term coefficients and the p-values are shown in Table 2. The coefficient of the model constant was 72.65, the standard error of the regression was 1.96 and the R^2^ value was 95.02%. The residuals normal probability plot, residuals histogram, and plots of residuals versus fitted value and run order are shown in Figure 8, the plots do not indicate any major problems with the model as the residuals are following a normal distribution and show no major trends with any of the other variables plotted. The slight negative trend of negative residuals in the last few runs were some additional extreme sets of conditions run at the end of the study to ensure the optimal solution had been found.

**Table 2:** Included terms, term coefficients and term p-values in optimisation model

**Linear**	**Coeff**	**p-value**	**Square**	**Coeff**	**p-value**	**Interaction**	**Coeff**	**p-value**
Gray_Low	4.81	0.000	GrayLow *GrayLow	−1.65	0.031	GrayLow* Smooth	−1.35	0.034
B_Low	2.61	0.000	B_Low *B_Low	−3.21	0.029	GrayLow *SE	3.66	0.000
Smooth	4.46	0.000	Smooth *Smooth	−6.05	0.000	B_Low *Smooth	3.25	0.029
SE	−11.34	0.000	SE*SE	−5.50	0.000	Smooth*SE	6.45	0.000

**Figure 9:**
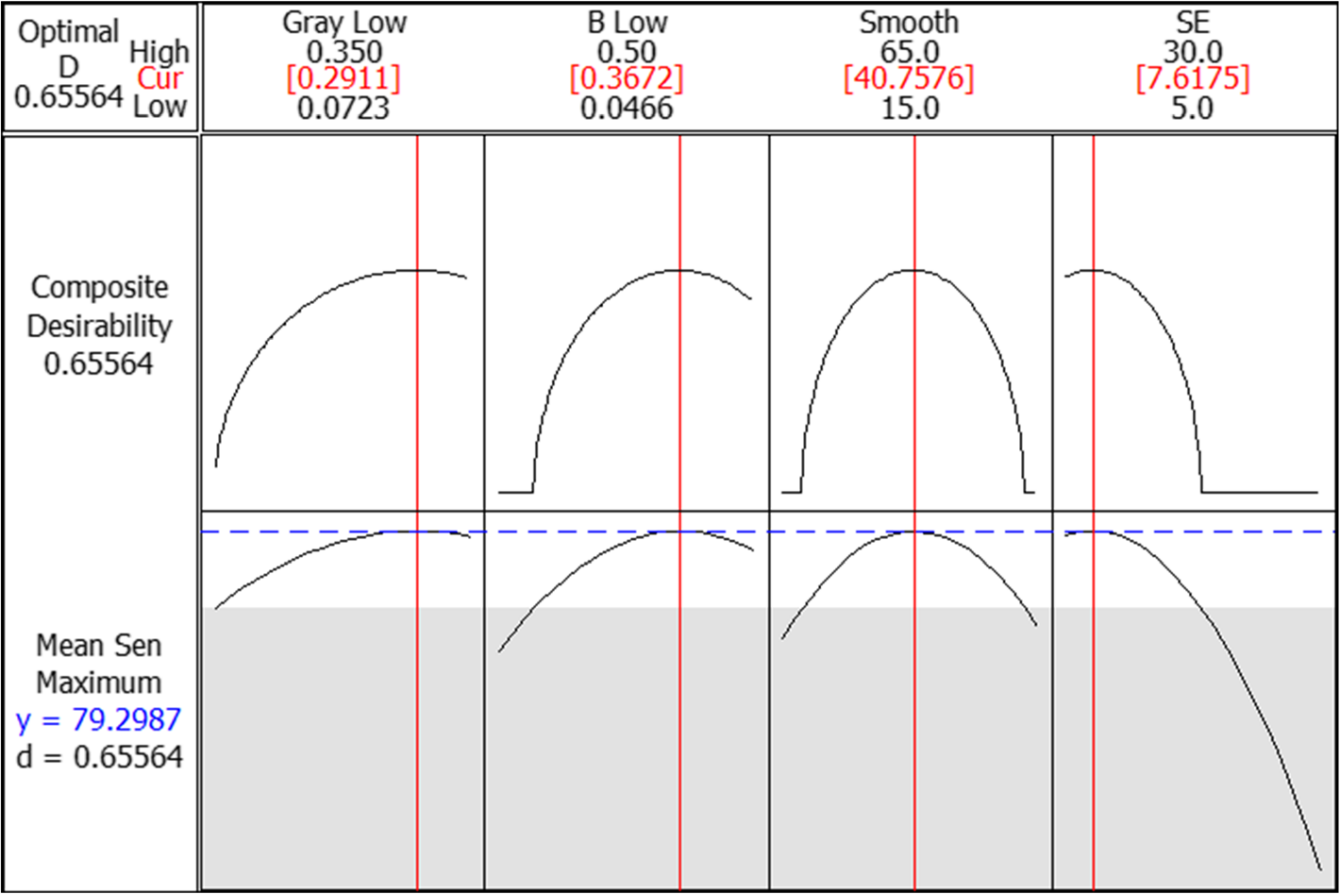
**Optimisation plot for key parameters to maximise sensitivity.** Figure 9 shows the effect of each parameter on sensitivity, and highlights the optimal values for each parameter. The suggested values are in square brackets above each column and the effect of varying each parameter on the mean sensitivity is shown in the plots below. The absolute value of the composite desirability value is not important as it is dependent on what target value and weight was set in the optimisation, and changing these values did not affect the optimisation result.

Once the model was built, the Response Optimiser in MINITAB was used to find the optimal set of parameters. This program works by employing a reduced gradient algorithm with multiple starting points to find the combination of input values for the parameters which maximise the mean sensitivity response. Mean sensitivity was used as the response in this case as the specificity was high for all the sets of conditions being tested. The optimal values are shown in Figure 9. The suggested values are in square brackets above each column and the effect of varying each parameter on the mean sensitivity is shown in the plots below. The absolute value of the composite desirability value is not important as it is dependent on what target value and weight was set in the optimisation, and changing these values did not affect the optimisation result. Since the high thresholds of both the grayscale and b* contrast enhancement had not been deemed significant in the model, they were set at the higher level of 1, as the slight effect they both had was to increase sensitivity when set at higher values. The final optimisation parameters resulted in a sensitivity of 76.4% and a specificity of 97.3% on the training set.

**Figure 10:**
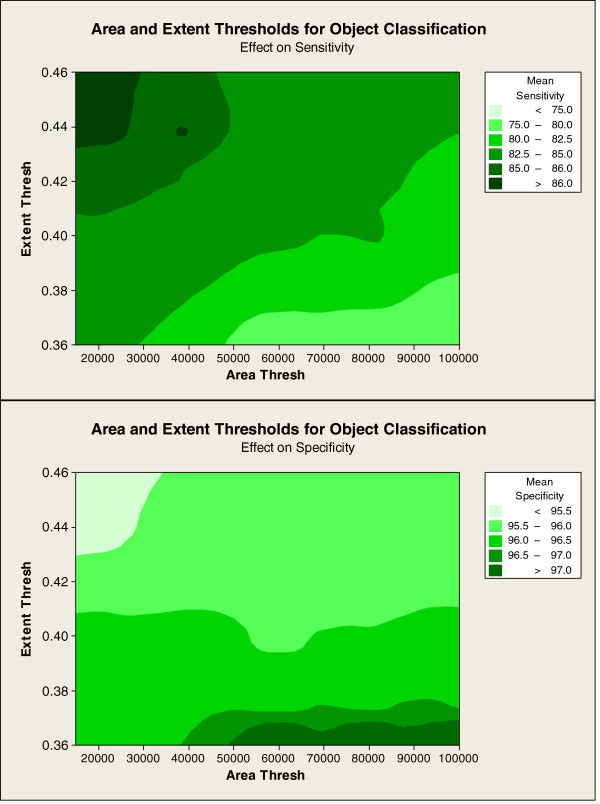
**Contour plot of the effect of area and extent object classification thresholds on mean sensitivity and specificity for segmentation of epidermis.** Figure 10 shows the results of an optimisation of area and extent threshold for object classification when the object pixel area threshold was varied between 15000 and 100000 pixels, and the extent threshold varied between 0.36 and 0.46. The top plot shows the effect on sensitivity and the bottom plot shows the effect on specificity. A distance based interpolation method, with a distance power of 2 was used to plot the contours.

The algorithm was further improved by optimising the object classification rules. Figure 10 shows the sensitivity and specificity of the final algorithm (without any user interaction, see method step 9), when the area threshold was varied between 15000 and 100000 pixels, and the extent threshold varied between 0.36 and 0.46. The values included in the optimisation were determined based on the typical extent and area of epidermis objects after the other processing steps set of 16 images. The surface plot is darker in colour when the sensitivity and specificity are higher; it was used to decide on an extent threshold of 0.44 and an area threshold of 20000, which balanced good sensitivity (86.9%) and specificity (95.3%) on the training set.

The optimised parameter values and final method were tested on the whole image set both with and without the user interaction step. It was clear that some of the worst performing images had unusual lighting or staining colour profiles. In attempt to address this issue, the colour normalisation step in the method was added. Without user interaction, the addition of this step improved the mean sensitivity of the training set from 86.9% to 91.8%, the mean specificity from 95.3% to 97.7% and the mean accuracy from 93.9% to 96.7%.

### Final algorithm performance evaluation

The optimised parameter values and final method were tested on the whole image set both with and without the user interaction step. Table 3 summarises the three performance metrics for the training set of 25 images, the test set of 15 images and the whole dataset in terms of the mean, standard error of the mean, maximum and minimum are given. The data is shown with and without the user interaction step.

**Table 3:** Summary of statistics for accuracy, sensitivity and specificity performance metrics

		**Training set (n = 25)**				**Test set (n = 15)**				**All (n = 40)**			
		**Mean**	**SEM**	**Max**	**Min**	**Mean**	**SEM**	**Max**	**Min**	**Mean**	**SEM**	**Max**	**Min**
**No user interaction**	**Sensitivity**	95.3	1.0	70.9	99.4	97.1	0.4	90.7	100.0	96.0	0.8	70.9	100.0
	**Specificity**	86.9	2.0	48.3	99.4	80.3	4.1	0.0	96.9	84.4	3.0	0.0	99.4
	**Accuracy**	93.9	1.1	71.6	98.6	94.3	1.0	72.7	98.7	94.0	1.0	71.6	98.7
**With user interaction**	**Sensitivity**	97.0	0.7	76.8	99.4	97.5	0.2	94.3	99.4	97.2	0.6	76.8	99.4
	**Specificity**	88.3	1.6	61.3	99.4	89.2	0.9	80.3	96.9	88.6	1.3	61.3	99.4
	**Accuracy**	95.6	0.8	77.2	99.0	96.4	0.3	92.7	99.0	95.9	0.7	77.2	99.0

Overall, the performance of the training and test set is fairly similar. Without user interaction, the training and test set mean specificities are the same (97.7%) and the training set accuracy is 0.7% higher than the test set accuracy (96.7% rather than 96.0%). There is a low variance in these metrics across the dataset, shown by the low standard errors (0.2-0.4%). There is more difference in the sensitivity metric, with a training set sensitivity of 91.8% and a test set sensitivity of 85.3%, and there is more variance in this metric across the data set, with standard errors of 1.3% and 2.2% for training and test set and a much larger range of values than are present in the other metrics. This variation is primarily due to a higher standard deviation within the data. When the user interaction step is added, the main effect is to reduce the variation in performance; for example the standard deviation of sensitivity for test set reduces from 14.2 to 8.4 when the user interaction step is added. Atypical images where the automated object classification rules have removed or retained objects incorrectly are more easily interpreted by a human operator, the addition of this step does not result in large improvements in the performance metrics, but it does significantly improve the segmentation of the poorest performing images.

**Figure 11:**
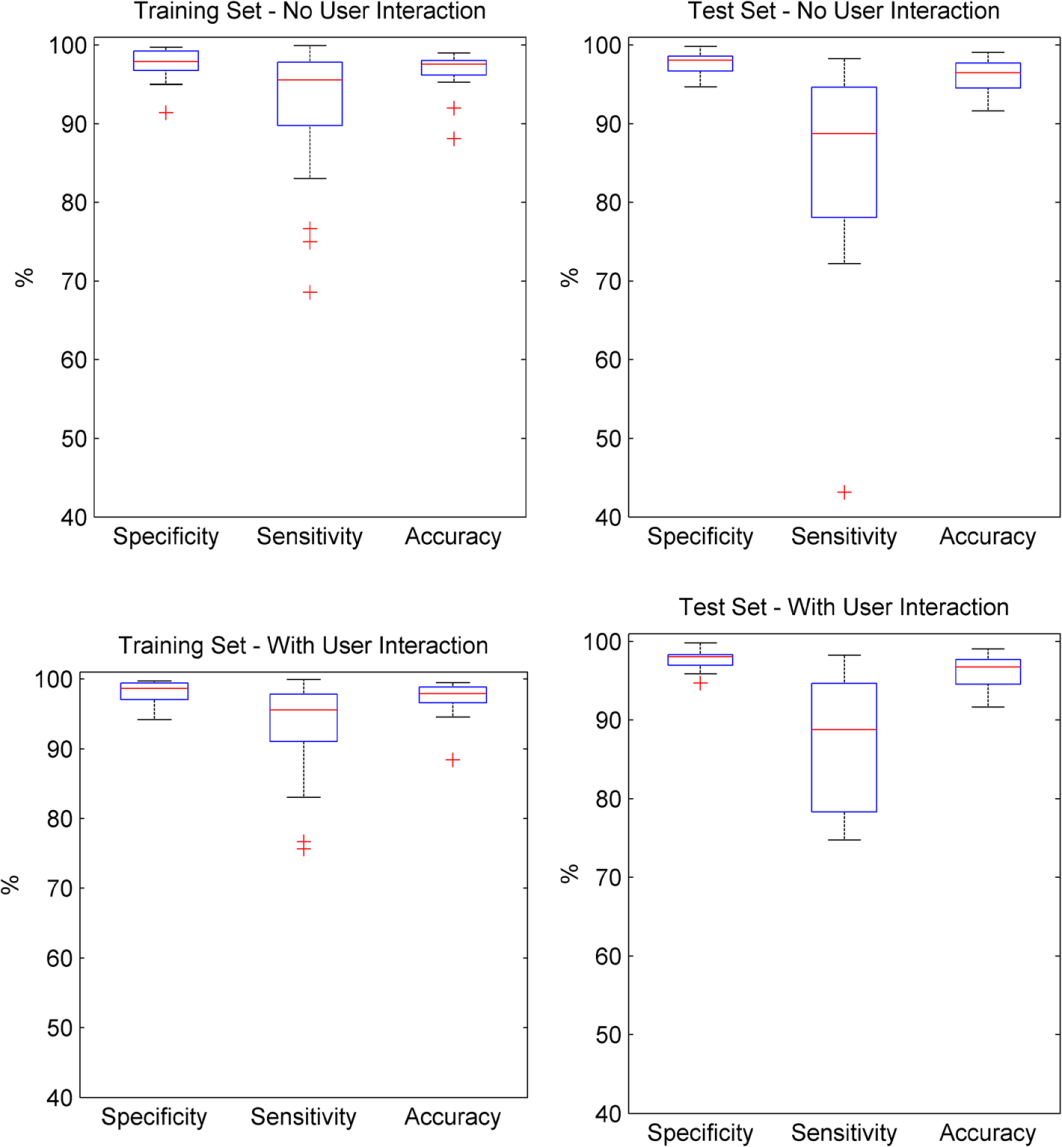
**Boxplot of specificity, sensitivity and accuracy for epidermal segmentation in training and test sets– with and without user interaction.** Figure 11 compares the specificity, sensitivity and accuracy metrics for epidermal segmentation of the optimisation training and test sets, with and without the addition of the user interaction step. The boxplot shows the minimum, lower quartile, median, upper quartile and maximum for each metric. Outliers are shown as individual crosses, with outlier status given if a point is lies more than 2.7 standard deviations from the mean, assuming a normal distribution.

A comparison of the training and test set data with and without user interaction is presented as a boxplot in Figure 11. The boxplot defines the median, interquartile range and highlights outlying results that are more than 2.7 standard deviations beyond the mean as crosses. The figure shows the similarity of performance in terms of specificity and accuracy for the training and test sets, with and without user interaction. The slight reduction in sensitivity of the test set can also be seen, as well as an increase in interquartile range and range compared to the training set. The user interaction step reduces the number of outliers with poor performance in the data.

Across the whole dataset of 40 images, including user interaction, the mean specificity is 98.0%, the mean sensitivity is 91.0% and the mean accuracy is 96.8%. Without user interaction, the mean specificity is 97.7%, the mean sensitivity is 89.4% and the mean accuracy is 96.5%.

Despite the variation in sensitivity for different images, the relatively low standard error suggests good confidence can be expressed in terms of the precision of the sample mean. The six worst segmentations in the dataset of 40 have sensitivities of 75-78% and they are not specific to a particular class of damage with two grade I, two grade II, one grade III and one grade IV.

**Figure 12:**
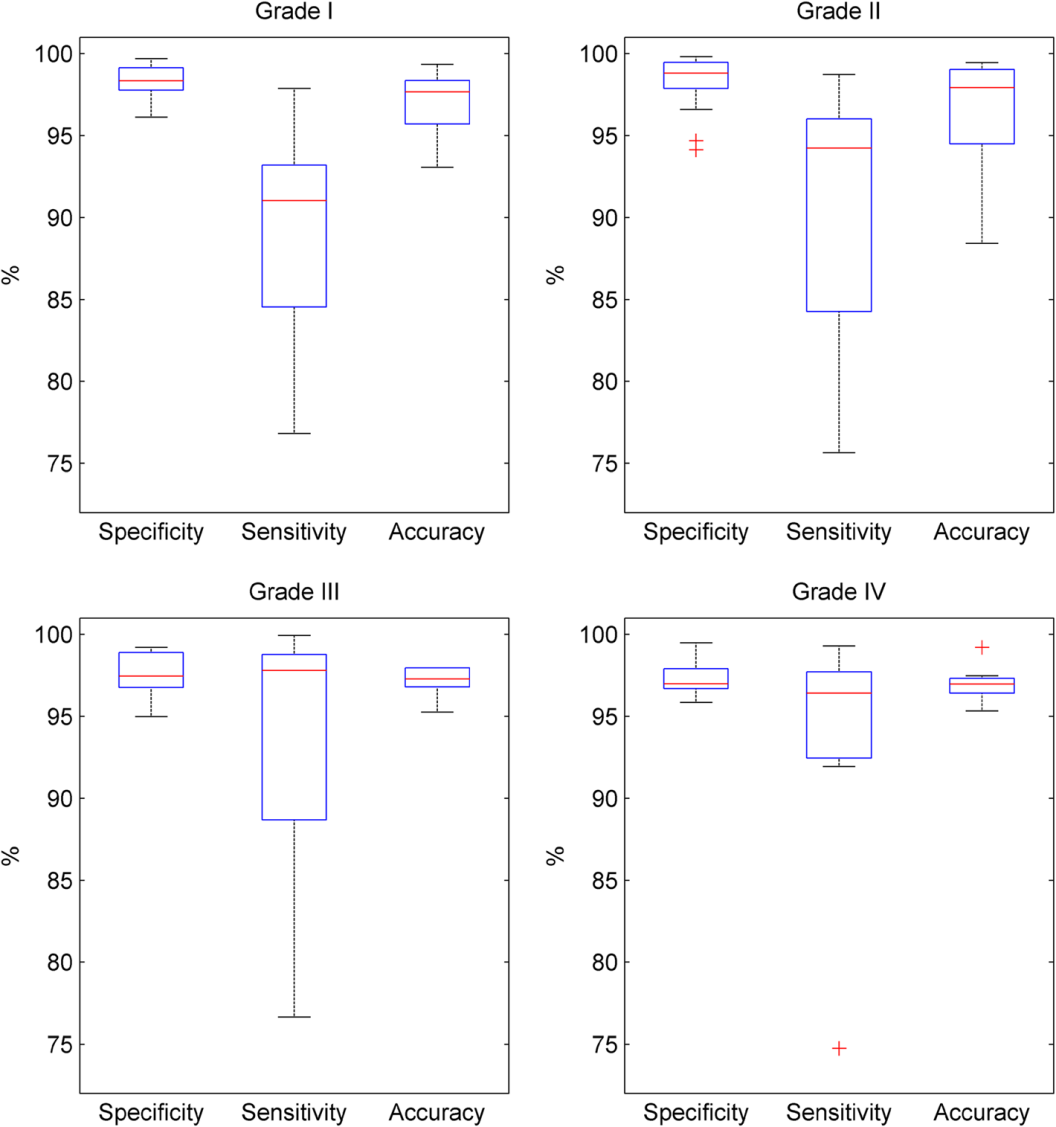
**Boxplot showing effect of damage grade on specificity, sensitivity and accuracy of epidermal segmentation.** Figure 12 shows the specificity, sensitivity and accuracy metrics for epidermal segmentation of the samples for each grade of damage. Each boxplot shows the minimum, lower quartile, median, upper quartile and maximum for each metric. Outliers are shown as individual crosses, with outlier status given if a point is lies more than 2.7 standard deviations from the mean, assuming a normal distribution.

Figure 12 shows individual boxplots of the data for each damage grade to ascertain whether certain grades of damage result in worse performance. From these results, it can be observed that there are small differences between the data, but no major differences. Considering the relatively small sample size once the data set is split into the four damage types, it is likely the changes seen are simply due to error within the small data set. The mean specificities are all between 97.3-98.3%, the mean sensitivities vary from 88.7-93.4% and the mean accuracies between 96.5-97.1%.

The algorithm (without user interaction) processes approximately 866,432 pixels per second with a standard deviation of 124,764. On average, it takes 11.4 seconds (with a standard deviation of 5.1) to process a typical image in this dataset using an Intel quad-core 3.4GHz processor with 8GB RAM.

## Discussion

The aim of this research was to develop an algorithm capable of segmenting the epidermis in images of H&E stained skin that exhibited varying degrees of histological damage. Epidermal segmentation is the critical first step in an automated procedure to detect and classify histological damage caused by immune responses within the skin. The developed method is robust in terms of its ability to segment the epidermis even in cases where the morphology and structure has broken down.

After image cropping and an initial segmentation of sample pixels to improve algorithm efficiency the main process can be applied. After a colour normalisation step based on histogram matching to a well stained target image in the RGB colour space, pixel colour and staining intensity information is captured through a linear combination of two image representations. Colour information relating to the staining is captured using a contrast enhanced b* plane from the L*a*b* colour space, and general staining intensity information is identified using a contrast enhanced grayscale image. The two image representations are mean filtered to remove some of the cellular detail within the different tissue types, then combined to maximise contrast between the epidermis and the rest of the skin tissue. Following Otsu thresholding, the segmentation is fine-tuned using morphological processing, and a final object classification step based on size and shape is applied.

The proposed method segments the epidermis from whole slide skin images with a mean specificity of 98.0%, a mean sensitivity of 91.0% and a mean accuracy of 96.8%, which is an improvement on previously published segmentation approaches for epithelial tissues such as those reported by Wang *et al.*[25], who achieved accuracies of 94.9 – 96.3%, and Eramian *et al.*[27] who achieved an accuracy of 85%, and mean sensitivity and specificity of 0.914 and 0.846 respectively. The balance of sensitivity and specificity required is dictated by the particular application. In this case, a low specificity caused by incorrect inclusion of dermis pixels or *stratum corneum* pixels in the epidermis segmentation results in an unacceptable number of false positive results in the feature extraction and classification process which follows the segmentation. Some reduction in sensitivity at this stage to allow increases in specificity is therefore acceptable in this application.

The time efficiency is difficult to compare accurately with other methods as it is dependent on the computer system used. However an approximate number of pixels processed per second can be used to compare methods. The 11.4 second average run time which processes ~866,432 pixels per second processed compares favourably with Eramian *et al.*[27] who quoted an average runtime of 7.2 s per image, which can be scaled to ~189,583 pixels per second. Wang *et al.*[25] were processing much larger images, so despite a reported runtime of 21 minutes, the pixel processing per second works out as ~ 7,619,048. Additional time efficiencies could be gained if the some of the more time consuming functions such as colour space conversions were translated to MEX-files.

A colour normalisation step was included prior to the implementation of the main segmentation algorithm to improve the robustness of the method, and enable it to cope with staining and lighting variation in the input images. The inclusion of a colour normalisation step is a trade-off between retaining as much colourimetric information as possible within the images and managing the variation resulting from sample preparation, staining and imaging. Mapping to an ideal target image can be problematic since each image has differing proportions of background to sample, and also of epidermis to dermis tissue. The effect of carrying out a mapping between differing images is that some differences are smoothed out, while others are enhanced. These effects were mitigated in this study by confining the colour mapping to sample pixels in the target and test images. The ability of the algorithm to achieve high accuracy, sensitivity and specificities despite significant variation in the input images shows the approach was effective. Applying colour normalisation prior to the colourspace conversion and contrast enhancement steps that follow ensures that the effects of staining and lighting variation in the input image are mitigated early in the process and prior to the following contrast enhancement and thresholding steps. The initial colour normalisation also enhances the contrast between epidermis and dermis in images where there is poor contrast between the two tissue types. This prior normalisation step enables the following contrast enhancement to be more finely tuned. Without the normalisation step, variations in overall colour hue, saturation and intensity (caused by staining and lighting differences) place significant pressure on subsequent processing steps leading to reduction in final segmentation accuracy and sensitivity.

Key parameters for the segmentation were optimised together, as there is significant interaction between them. For example, the smoothing operation of the grayscale and b* images described in step 4 of the method affects the scale and resolution of variation within the image, and therefore impacts on the size of structuring element required to fine tune the thresholding. Once these parameters were optimised, the method was robust enough to work effectively on most of the images in the dataset.

The algorithm as presented here has applications beyond the grading of adverse immune reactions, and is a useful framework on which to build any skin segmentation, such as epidermal segmentation prior to epidermal thickness measurements, detection of melanoma, or diagnosis of dermatological conditions such as psoriasis. Furthermore the approach could be applied to other types of tissue, in particular other epithelial tissues with H&E staining. The critical parameters optimised in the Design of Experiments study may need to be altered slightly for different tissues, and the sensitivity could be increased (at the expense of specificity) using the object classification step.

## Conclusions

The main contribution of this work is the development of a new methodology for segmentation of epidermal tissue, an area of research where there are few published methods. The addition of a pre-thresholding colour normalisation and contrast enhancement protocol and a post thresholding morphological processing step enable the traditional and easily implemented Otsu thresholding algorithm to be applied to challenging histopathological datasets.

The robustness is shown by the method’s high accuracy in segmentation of a challenging dataset of epidermis tissue from H&E images of human skin showing varying degrees of histological damage. The proposed method segments the epidermis from whole slide skin images with a mean specificity of 98.0%, a mean sensitivity of 91.0% and a mean accuracy of 96.8%.

By adjusting the pre-threshold smoothing and post-threshold morphological processing parameters and the object classification rule, the methodology could be optimised for other H&E stained tissue segmentations. The same parameters could also be adjusted to apply the method for images of different magnification and resolution.

## Competing interests

The authors declare that they have no competing interests.

## Authors’ contributions

JH implemented the algorithms presented in the manuscript, tested the algorithms, and prepared the first draft of the manuscript. EBM participated in the design and coordination of the study, advised on the analysis of the results and revised the manuscript. AD and XNW proposed the project, advised on the requirements of the algorithm, prepared the skin explant slides, advised on the selection of samples and revised the manuscript. CO contributed to the analysis of the results, implementation of the algorithm and revised the manuscript. All authors read and approved the final manuscript.

## Pre-publication history

The pre-publication history for this paper can be accessed here:

http://www.biomedcentral.com/1471-2342/14/7/prepub
